# Evaluation of an intervention to support decisions on disclosure in the employment setting (DECIDES): study protocol of a longitudinal cluster-randomized controlled trial

**DOI:** 10.1186/s13063-020-04376-1

**Published:** 2020-05-29

**Authors:** K. M. E. Janssens, J. van Weeghel, C. Henderson, M. C. W. Joosen, E. P. M. Brouwers

**Affiliations:** 1 Tranzo, Tilburg School of Social and Behavioral Sciences, Tilburg University, Tilburg, The Netherlands; 2 Kenniscentrum Phrenos, Utrecht, The Netherlands; 3 Department of Health Services and Population Research, King’s College London, London, UK; 4 Department Human Resource Studies, Tilburg School of Social and Behavioral Sciences, Tilburg University, Tilburg, The Netherlands

**Keywords:** Mental health issues/illness, Unemployed people, Employment specialists, Disclosure, Employment

## Abstract

**Background:**

Unemployment rates are higher among people with mental health issues/illness (MHI) than in the general working population, and many of them face the dilemma of whether or not to disclose their MHI when searching for employment. Disclosure can lead to rejection and discrimination, but alternatively can also have important advantages that may be necessary to retain employment. Whether disclosure decisions lead to sustainable employment depends on many factors, of which unemployed people themselves can only influence their decision to disclose or not and the way in which they communicate. This study evaluates the cost-effectiveness of an intervention to support unemployed people with MHI in their disclosure decision and communication.

**Methods:**

This is a two-armed, clustered, randomized controlled trial with longitudinal design and randomization at organization level. An intervention will be examined, which consists of a disclosure decision aid tool (CORAL.NL) for unemployed people and workplace stigma-awareness training especially designed for employment specialists, which focusses on how to support unemployed people in their disclosure decisions. Participants in the intervention group are unemployed people who receive support from trained employment specialists from organizations allocated to the intervention group, and receive the CORAL.NL decision aid after baseline. The control group consists of unemployed people who receive support as usual from employment specialists from different organizations allocated to the control group. Primary outcomes are: cost-effectiveness of the intervention, e.g. healthcare costs, having employment, days until start of employment, independency of social security, having other forms of employment and decision making about disclosing MHI. Secondary outcomes are mental health and wellbeing, stigma and discrimination and work-related factors. Financial income data are collected via the registration systems of Dutch municipalities and Statistics Netherlands, and by questionnaires at baseline, and at 3, 6 and 12 months.

**Discussion:**

If using a decision aid to decide about disclosure of MHI leads to people finding and retaining employment more often, this study will contribute to lowering healthcare and societal costs.

**Trial registration:**

Netherlands Trial Register: NL7798. Registered on 4 June 2019.

## Introduction

People with mental health issues/illness (MHI) are more often unemployed than people without MHI [1–4]. In addition, people with MHI who are employed have higher risk of losing their employment [3, 5] and increased risk of dropping out of work, due to unemployment, work disability, long-term absenteeism or early retirement [6]. Studies have shown that unemployment exacerbates MHI [7] and that when people with MHI start working again, this positively affects their mental health [8].

One of the barriers to people with MHI finding and retaining employment is a negative attitude towards MHI. Multiple studies have shown that the stigma attached to MHI is a risk for not entering the job market or not returning to existing employment [9, 10]. There are several reasons why stigma is a problem for employment, e.g. many employers have negative attitudes towards people with MHI [11–13], which often has negative effects for people with MHI in job applications, contract extensions, job promotions and other career opportunities. Moreover, anticipated discrimination (e.g. avoiding situations or activities because of the fear of being discriminated) and self-stigma (e.g. having negative ideas about oneself because of the MHI) can lead to feeling one is not performing well and therefore had better not try anything [14]. This “why try-effect” discourages people from engaging in relevant activities, such as applying for jobs [15]. International studies have shown that large numbers of people (39-64%) with depression, addiction problems or schizophrenia refrain from applying for jobs or receiving training or education because of possible reactions of others [9, 15, 16]. Furthermore, employees with MHI often do not feel comfortable in talking about their MHI. As a result, employers and employees miss out on the opportunity to talk about the need for support and (temporary) work adjustments. This is unfavorable, because work accommodations, such as adjustment of working hours, can prevent and reduce absenteeism [17].

As a result of stigma, whether or not to disclose MHI in the workplace is a major dilemma for many people with MHI of working age. Disclosure can lead to better work outcomes (i.e. due to appropriate work adjustments), but also to not being hired [18]. The decision whether or not to disclose is often perceived as stressful [19, 20] in which advantages and disadvantages are weighed against each other. In 2010, the Conceal or reveal (CORAL) decision aid was developed by researchers at the Institute of Psychiatry at King’s College London [21]. The purpose of this decision aid is to support decision making about disclosure in the work context [22]. The principle of this decision aid is that people know their own situation best and therefore can make the best choices themselves, but still benefit from help with making a choice. In several follow-up studies [23, 24], using the decision aid was found to be promising: people who used CORAL had less decision-making stress and were significantly more often working full time after 3 months than people who did not use the decision aid [24]. Recently, a randomized controlled trial (RCT) was conducted using a web-based decision aid tool (READY) to help facilitate people in current employment in making disclosure decisions about mental health conditions [25]. Participants who used READY had significantly less decisional conflict about disclosure of a mental health condition and were at a later stage of decision making. These results are very promising for disclosure decisions in the employment setting and would potentially be relevant to implementing and evaluating a similar decision aid tool for unemployed people with MHI in a different context in the Netherlands.

### Objective and research questions

An RCT is conducted to examine the effects of an innovative intervention based on the English CORAL decision aid [23], which has been adapted to the Dutch context and embedded in an intervention for unemployed people with MHI and workplace stigma-awareness training especially designed for employment specialists. Furthermore, factors that facilitate finding employment and factors that hamper this will be studied. The primary research questions of this study are:

1. Does the intervention more often lead to finding and retaining paid employment for unemployed people with mental health problems, compared to usual guidance in municipal practice, controlled for other factors (e.g. mental health and stigma and discrimination)?
2. Is the intervention cost-effective from a societal perspective (including reintegration costs and healthcare costs)?
3. For whom, under which circumstances and in what way does the intervention work best or less well, and why?

## Methods

The Consolidated Standards of Reporting Trials (CONSORT) 2010 statement and Standard Protocol Items: Recommendation for Interventional Trials (SPIRIT) 2013 statement were followed in the design of the study [26, 27]. The study is funded by The Netherlands Organization for Health Research and Development (project code 535001003). The Ethic Review Board of Tilburg University approved the study design, protocol, information letter, informed consent form and the questionnaires (EC-2018.06 t). The study is registered under trial registration number NL7798.

### Study design

**Fig. 1:**
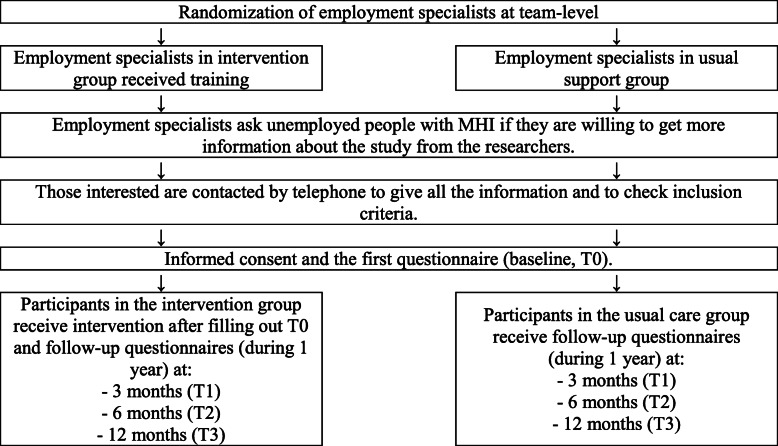
Flow diagram of the study protocol. MHI, mental health issues/illness

The DECIDES study is a longitudinal, two-armed, clustered RCT of unemployed people with MHI who receive social benefits and/or reintegration support from Dutch municipalities. In this RCT the effects of an intervention that consists of a decision aid for unemployed people (CORAL.NL) and training for employment specialists who guide them in their job-seeking process are evaluated. Randomization took place at organization level (see Fig. 1). Participants are assessed at baseline (T0), and at 3 months (T1), 6 months (T2) and 12 months (T3). In addition, data on employment history (e.g. having employment, income, working hours per week and employment characteristics such as contract and employment type) and social benefits (e.g. having social benefits, duration of social benefits and the amount of social benefits) are extracted anonymously from the registration systems of the municipalities and Statistics Netherlands from T0 to T3 in participants who give consent for this. Collecting data from registration systems is more reliable and is less burdensome for participants. Participation in the study is voluntary and all participants sign an informed consent form for participation, and provide separate consent for the retrieval of their personal data from Statistics Netherlands. Measurements take place in one-by-one appointments with a researcher on the project. Participants can fill out the questionnaire digitally or by paper and pencil. If necessary, the researcher gives support by filling out the questionnaire, e.g. by explaining or reading out loud the questions for illiterate participants. Participants were stimulated to complete follow up by handing out a financial remuneration of 10 euros and by asking several contact options (mobile phone, email) to maintain contact during the participation period. If participants give consent to collect their data from Statistics Netherlands these data will also be collected if they discontinue filling out the questionnaires.

### Setting

In the Netherlands, people older than 18 years are entitled to social benefits if they have insufficient income or capital and are unable to make use of another provision or benefits, such as disability benefits. In order to receive social benefits, (re)integration obligations must be met, such as cooperating in the support that the municipality offers aimed at entering the job market or returning to existing employment. This support is offered per municipality, and is often organized differently per municipality. Regarding disabilities and employment, the Netherlands has confirmed the U.N. Convention on the Rights of Persons with Disabilities [28] and has its own Disability Discrimination Act. The convention and act indicate that organizations and employers need to ensure that employees with disabilities have access to reasonable accommodations at work [29]. This anti-discrimination legislation may influence the employment status of people with disabilities in various ways. Employees do not have legal obligations to inform the employer about a disability as long as the impairment does not result in any endangerments at the workplace. However, disclosure of a health problem may be necessary for access to accommodations whereby this only can be implemented if the employer has knowledge of the disability, especially when natural support in the workplace is not available. Organizations commonly perceive such legislation and policies as a burden, e.g. because Dutch employers must pay at least 70% of the salary of a sick employee during the first 2 years of sickness absence [30]. This in fact might lead employers to try to avoid hiring a person with a disability [29].

### Intervention

The CORAL decision aid was originally developed in the UK [21, 23, 24] and was first tested in 2013. In that RCT, using CORAL among unemployed people with MHI more often led to full-time employment and less decisional conflict than in the control group [24]. The current study examines the effects of the CORAL decision aid in the Netherlands. For this study, CORAL has been modified to provide a newer version for the Dutch context, CORAL.NL, and has been extended with two infographics and newly developed training targeted at employment specialists.

### CORAL.NL

In 2017, prior to this study, the CORAL decision aid was translated and developed further to fit into Dutch practice. To attain this, focus groups were held with (1) people with MHI, (2) employers, (3) human resource managers, (4) mental health advocates and (5) employment specialists [18]. The new CORAL.NL decision aid was tested and implemented in pilot tests. Contrary to the original UK CORAL decision aid, which is designed for independent use, the Dutch CORAL.NL decision aid is a comprehensive module in which people with MHI and their employment specialists are able to discuss disclosure of MHI in the work context, so that informed decisions can be made and implemented. CORAL.NL consists of four parts with several paragraphs: Part 1 deals with choices about disclosure and contains the pros and cons of disclosure and the personal disclosure needs and values. Part 2 is about one’s personal situation and deals with questions about when and to whom disclose should be made. Parts 3 and 4 summarize previous sections to make a plan about whether to disclose or not, and if so, to whom and when and what to disclose. In addition to CORAL.NL, for this study two one-page infographics have been developed that summarize the most important information from CORAL.NL: one version about disclosure during the job application process and one version about disclosure in the work context for people who already have employment. These infographics provide an easy to read one-page summary of the CORAL.NL booklet and were developed because during a pilot test, some respondents found it difficult to use the CORAL.NL booklet itself as they had trouble reading or concentrating.

### Intervention/training-based care

Employment specialists who are allocated to the intervention group receive workplace stigma-awareness training about disclosure of MHI in the work context, from the start of this study. This training is specifically designed for the purpose of this study and consists of three meetings within 6 months. Each meeting has a duration of 2 h and is provided in groups of 4–12 employment specialists under guidance of 2–3 trainers. The aim of the training is to enhance awareness of stigma, discrimination and the disclosure dilemma and to introduce the CORAL.NL tools (including the booklet and infographics). Factors that contribute to reducing stigma and discrimination using training interventions are education and social contact between people with and without MHI [31]. Therefore, informative presentations are given during the training sessions, people with lived experiences are present, a film is shown in which people with lived experienced share their experiences and feelings about stigma and discrimination in the work context, discussions take place and using role-play, employment specialists have the opportunity to practice what they learn. After the first meeting, employment specialists have the skills to use the CORAL.NL tools. Several aims are addressed: (1) creating awareness of stigma and discrimination in the work environment by providing insight into what stigma is, how it works and how it can be experienced and what the effects of stigma are; and increasing insight into stigma and discrimination by employers and managers, the effects of employment specialists’ own attitudes, personal prejudices and actions and to increase insight into the negative effects of disclosure in job applications; (2) increasing understanding of how the disclosure dilemma can be experienced by people with MHI, how it affects people and how the conversation about disclosure can be started, without influencing the outcome and (3) learning to work with the CORAL.NL tools, including how they can be used in daily practice and experiences of working with the tools. Employment specialists are stimulated and reminded to use CORAL.NL after participants have completed T0 (baseline).

### Support as usual

Participants in the control group receive support as usual from their employment specialists. Neither participants nor employment specialists are introduced to CORAL.NL. In the Netherlands, people who receive social benefits from their municipality, have the responsibility to (re-)integrate into employment. Municipalities offer various facilities such as guidance from employment specialists, education and training.

### Procedure

#### Randomization of employment specialists (ES)

All 72 participating employment specialists were recruited between November 2017 and March 2018 from eight participating organizations. Two researchers presented the study during meetings at the local organizations, provided written information about the study and provided a registration form and informed consent form. After including all employment specialists, the organizations were randomly allocated to either the intervention or control condition, using SPSS software. Cluster randomization was chosen, as individual randomization would have higher risk of contamination between the intervention and control group, because employment specialists within organizations work together on a daily basis. Due to the nature of the intervention, neither the employment specialists nor the researchers can be masked to the allocation to the conditions.

#### Recruitment of participants

Participants are recruited via the employment specialists working at eight different organizations, and via newspapers and personal letters from the organizations. Inclusion criteria for the study are (1) being unemployed, (2) having sought any treatment (currently or in the past) for MHI, including addiction, by a health professional (e.g. general practitioner (GP), psychologist) and (3) adequate command of the Dutch language, as the intervention and questionnaires are in Dutch. Employment specialists are asked to provide people who meet the inclusion criteria with information about the research and to ask if they are willing to receive more information about the research. If participants give permission to share their contact details with the researchers, they are informed about the research by telephone and the inclusion criteria are checked.

### Outcomes

Table 1 presents an overview of the collected data and the study time path.

**Table 1:** Data collection and time path

Topic	Instrument	Baseline	Follow up		
		T0	T1	T2	T3
			3 months	6 months	12 months
**Primary outcomes**					
Cost-effectiveness of the intervention	TiC-P EQ-5D-5 L	X	X	X	X
Having employment (yes/no)	Data from Statistics Netherlands^a^ Municipal administration Questions about work, income and benefits	X	X	X	X
Days from baseline until start employment (*n*)		X	X	X	X
Receives social benefits (yes/no)		X	X	X	X
Having other forms of employment (i.e. voluntary work)		X	X	X	X
Decision making about disclosing MHI	DCS Stage of decision making	X	X	X	X
**Secondary outcomes**					
Mental health	PHQ	X	X	X	X
Wellbeing	WEMWS	X	X	X	X
Stigma	ISMI-10	X	X	X	X
Discrimination	DISC (shortened version)	X	X	X	X
Work-related factors	Job seeking activities Personal fears about getting to work RTW-SE PSWOHP (shortened version)	X	X	X	X
**Prognostic measures**					
Age, gender, nationality, marital status, level of education		X			
History of mental ill-health		X	X	X	X
Characteristics of work and/or social benefits		X	X	X	X
**Additional measures**					
Work values/core capabilities	Core Capability Set	X	X	X	X
Characteristics of employment specialists	Age, education, years of work experience of employment specialists OMS-HCP	X			X
Personal experiences and satisfaction with CORAL.NL^b^	Questions about the use of the decision aid Interviews with employment specialists and participants in intervention group	X	X	X	X

*TiC-P* Treatment Inventory Costs in Psychiatric Patients, *EQ-5D-5 L* Euroqol-5 dimensions-5 levels, *DCS* Decisional Conflict Scale, *MHI* mental health issue/illness, *PHQ* Patient Health Questionnaire, *WEMWS* Warwick-Edinburgh Mental Wellbeing Scale, *ISMI-10* Internalized Stigma of Mental Illness, *DISC* Discrimination and Stigma Scale, *RTW-SE* Return To Work Self Efficacy Scale, *PSWOHP* Patient Satisfaction With Occupational Health Professionals scale *OMS-HCP* Opening Minds Scale for Healthcare Providers

^a^ If participants agree with access to personal data from Statistics Netherlands, data are also collected from baseline to T3 if they discontinue to fill out the questionnaire

^b^ Only for participants in the intervention group

#### Primary outcomes

The primary outcomes of this study are (1) cost-effectiveness, which will be measured from a societal perspective comparing the intervention with usual care. Healthcare utilization and loss of production will be measured using the Treatment Inventory Costs in Psychiatric Patients (TiC-P), which is a reliable instrument with satisfactory validation [32]. The primary cost-effectiveness outcomes are having employment (yes/no), days from baseline until start employment, receiving social benefits (yes/no) and/or having other forms of employment (i.e. voluntary work, internship). The secondary cost-effectiveness outcome is the EuroQol-5D-5 L, which measures health-related quality of life [33], and (2) decision making about disclosing MHI, measured using the Decisional Conflict Scale, which has adequate test-retest reliability [34], and stage of decision making [35].

#### Secondary outcomes

Secondary outcomes will be explored as follows:

- Mental health is measured using the Dutch version of the Patient Health Questionnaire [36–38], which is used to measure the most common psychological diagnoses (mood disorders, anxiety disorders, alcohol abuse, somatoform disorders and eating disorders), and has good diagnostic validity [36].
- Wellbeing is measured using the Warwick-Edinburgh Mental Wellbeing Scale [39], which measures positive mental wellbeing and has good content validity and test-retest reliability.
- Stigma is measured using the brief Internalized Stigma of Mental Illness Scale-10 [40], which measures self-stigma among people with MHI and has good internal consistency.
- Discrimination is measured using two items of the Discrimination and Stigma Scale [15], which focuses on discrimination when finding and keeping employment.
- Work-related factors such as job-seeking activities are measured using four items, e.g. “Have you applied to a job vacancy in the last four weeks?”; personal fears about getting to work are measured using five items with a 5-point Likert scale, e.g. “Because of my mental health issues/illness, I have less opportunities finding employment”; work-related self-efficacy is measured using the Return to Work Self-Efficacy scale [41], which has good internal consistency and adequate test-retest reliability; and finally, the quality of guidance from employment specialists is measured using three items of the Patient Satisfaction With Occupational Health Professionals scale [42].

### Prognostic measures

The following prognostic data will be collected: personal characteristics such as age, gender, nationality, marital status, level of education and history of mental and physical ill-health.

### Additional measures

A variety of factors that can be influenced by the intervention or can affect the chances of finding employment are also measured:

- Core capabilities are measured using the Core Capability Set [43], which assesses the capabilities that are important for individuals (what they value) in relation to employment: this is a validated measurement. An adapted version of the Core Capability Set is used for participants without employment.
- Employment specialists receive two short questionnaires, i.e. at the beginning of the research and after including all participants, which contains questions about their demographics (age, education, years of work experience), experiences with people with MHI and attitudes towards people with MHI, measured using the Opening Minds Scale for Health Care Providers [44], which has good internal consistency and satisfactory test-retest reliability.
- Process evaluation: experiences with the intervention are measured among participants in the intervention group. Questions focus on whether the decision aid has been used in recent months and participants’ opinions on the decision aid. Participants in the control group are asked at measurement T3 if they are familiar with the decision aid and if so, how they get familiar with using the decision aid and what their experiences are with the decision aid. Additionally, individual 1-h interviews are held with employment specialists and participants of the intervention group after the quantitative data collection. The interviews focus on for whom, under which circumstances and in what way the CORAL.NL decision aid work best or less well and why.

### Sample size

The power calculation is based on data from a recent international study on individual placement and support, which is an evidence-based reintegration model that is also used for people with MHI who want to have regular employment [45] and has the same primary outcome measure, i.e. obtaining employment. In this study, the average percentage employment was 50% in the intervention group and 20% in the control group [45]. Considering 50% and 20% as possible percentages, 36 unemployed people are needed in each group to find a statistically significant difference (with a 5% significance level and power of 80%): 47 participants per group would be needed for power of 90%. However, in this study any cluster effects and the expected dropout of participants over the four measurements must be considered. Considering a dropout rate of approximately 40% because of the vulnerable population, a safe assumption is to have 75 participants per group, which means a total of 150 participants.

### Statistical analysis

Data will be processed using the statistical software SPSS. Data in this trial will be analyzed on the basis of the statistical principle “intention to treat”, i.e. participants will be analyzed in the arms to which they are assigned. Descriptive analyses will be used to detect significant differences in the baseline characteristics between the intervention group and control group. Longitudinal multilevel analysis will be used to analyze the outcomes. Subgroup analyses will be performed on baseline characteristics and decisional stress at baseline to test whether the groups differ based on baseline characteristics. No additional adjusted analyses will be performed. Baseline characteristics of participants with and without missing values will be examined to test for bias due to missing data. Classical methods of multiple imputations will be used for missing data.

## Discussion

In the past, the biomedical model was predominant in research in the field of medicine and healthcare, and psychosocial factors were under-investigated [46]. Nowadays, there is more evidence that psychosocial factors such as stigma and discrimination are of major influence in relation to employment for people with MHI [9, 18, 46, 47]. This study provides insight into the effects of unemployment and finding employment on the health and wellbeing of people with MHI and is one of the first studies to investigate the cost-effectiveness of an innovative decision aid tool for making decisions about disclosure of MHI in the workplace. Previous research has shown promising effects on finding and obtaining work using a decision aid for disclosure of MHI [24, 48]. Besides that, evidence suggests that adequate preparation of MHI disclosure decisions is of crucial importance in finding and keeping employment [18]. The societal relevance of this study is that the CORAL.NL decision tool could represent substantial healthcare and societal savings if it is effective in helping unemployed people with MHI to find and remain in employment.

### Strengths and limitations

A strength of this study is the collaboration with eight field organizations, mostly municipalities, in the Netherlands. Because each municipality organizes its employment services differently, this study is a representation of actual Dutch practice, yielding a heterogeneous population that allows generalization of the results to a larger population. Also, data from the questionnaires are combined with data from the register data from the Dutch municipalities and Statistics Netherlands, which gives the opportunity to collect very objective, reliable and detailed data. Limitations of the study are that participants are recruited via employment specialists, which may cause selection bias from the individual employment specialists, and participation in the study is entirely voluntary, which increases the risk of early dropout.

### Impact of study results

This study will show whether using the intervention leads to unemployed people with MHI finding and retaining employment more often, and to less decisional stress about disclosing MHI. If the intervention is cost-effective, this study will also contribute to lower healthcare and societal costs and fewer people with MHI who remain unemployed. Findings will be disseminated through peer-reviewed international and national publications and international and national conference presentations. Publications will be actively disseminated to all relevant groups via social media and through the Sponsor. A national symposium will be organized at the end of the research project. Results of this study will become available in 2021.

## Trial status

The study is registered under trial registration number NL7798 (registered 4 June 2019 - retrospectively registered; https://www.trialregister.nl/trial/7798). Participant recruitment started in April 2018 and ended in July 2019. Data collection will end in July 2020. The data gathering is in progress. According to the municipalities, there were difficulties in engaging and scheduling appointments with the target group, which delayed the submission of the study protocol paper. Priority was given to the hundreds of face-to-face meetings that need to be scheduled for data gathering. However, the researchers have no access to the primary outcome measures of the study until 3 months after the final measurement, as the primary outcome data will be retrieved from a different organization (i.e. Statistics Netherlands, an organization with very strict data security) 3 months after the end of the data gathering process.

## Data Availability

The (anonymized) datasets obtained during the current study are available from the corresponding author on reasonable request. The data will be pseudonymized, i.e. participants will be allocated an individual trial identification number. The data and the associated key file will be securely stored (separately from each other) on a Tilburg University secure network drive to which only the executive researcher, project manager and quality officer have access, in accordance with the University data policy. Paper versions of the signed informed consent forms and questionnaires will be scanned and securely stored on the Tilburg University secure network drive. After that, the paper version will be destroyed.

## Abbreviations

MHI: Mental health issues/illness
CORAL: Conceal or reveal
RCT: Randomized controlled trial
